# Phosphorescent Molecularly Doped Light-Emitting Diodes with Blended Polymer Host and Wide Emission Spectra

**DOI:** 10.1155/2013/954146

**Published:** 2013-11-13

**Authors:** Jun Wang, Jun Gou, Weizhi Li

**Affiliations:** State Key Lab of Electronic Thin Films and Integrated Devices, School of Optoelectronic Information, University of Electronic Science and Technology of China (UESTC), Chengdu 610054, China

## Abstract

Stable green light emission and high efficiency organic devices with three polymer layers were fabricated using bis[2-(4′-tert-butylphenyl)-1-phenyl-1*H*-benzoimidazole-*N*,C^2^′] iridium(III) (acetylacetonate) doped in blended host materials. The 1 wt% doping concentration showed maximum luminance of 7841 cd/cm^2^ at 25.6 V and maximum current efficiency of 9.95 cd/A at 17.2 V. The electroluminescence spectra of devices indicated two main peaks at 522 nm and 554 nm coming from phosphor dye and a full width at half maximum (FWHM) of 116 nm. The characteristics of using blended host, doping iridium complex, emission spectrum, and power efficiency of organic devices were investigated.

## 1. Introduction

Organic light-emitting diodes (OLEDs) have attracted more and more attention for their various potential applications such as display, backlight for liquid crystal display, and next-generation light sources since Tang and Vanslyke reported the bright green OLEDs with sandwiched structure [1–3]. Most phosphorescent OLEDs (PhOLEDs) typically were made up of a thin light emitting layer sandwiched between electron and hole blocking layers as well as charge transport layers by means of high vacuum evaporation techniques [4, 5]. The use of these multilayer architectures by vacuum deposition is expected to pose a challenge in reducing device manufacturing costs and controlling the doping concentration precisely. In contrast, solution processing of polymer-based organic semiconductors offers the potential for fabrication of electronic devices at a significantly reduced cost. Whether by spin coating or by ink jet printing, a variety of efforts have enabled high performance OLEDs to be commercialized [6, 7]. Burroughes reported the polymer organic light emitting diodes (PLEDs) using poly(*p*-phenylene vinylene) (PPV) as emission layer between indium-tin oxide (ITO) anode and metal cathode [8]. Polyvinylcarbazol film doped with dyes of coumarin 6, coumarin 47, and Nile red were used to fabricate PLED with ink-jet printing method by Hebner et al. [9]; the characteristics of the printing device were similar to the film with same composition deposited by spin coating method. Multicolor polymer light-emitting devices with solution processing have also been explored with cross-linked photochemically characteristic materials [10, 11].

But solution processed polymer light emitting devices are typically made up of not more than three layers, in which one of the layers performed the simultaneous tasks of charge transport and light emission [12]. Most of conjugated polymers typically indicate hole-only or electron-only transporting characteristic, which cause the unbalance holes and electrons in the emission layer. The unbalance charge within the device is unsuitable for improving power efficiency of PLEDs. Therefore, to achieve high efficiency PLEDs, several factors need to be considered, including the balance of electrons and holes, strong radiative transitions for singlet excitons, efficient light extraction, and developing phosphorescence emitters with triplet-triplet energy transfer characteristic.

Nonconjugated polymer poly(*N*-vinylcarbazole) (PVK) has been a commonly used polymer host for phosphorescent dyes [13–15]. But PVK has, however, an inherent defect in that its electron and hole mobility difference are too large [16]. In contrast to PVK-hosted PLEDs, the performances of PFO-hosted PLEDs could be enhanced from the selective removal of the electron-transporting material during fabrication of the functional layer [17], so that using PVK as hole transporting layer and PFO blended host layer is more suitable for high efficiency polymer device.

In this study polymer organic devices with three polymer layers structure including hole injection layer, hole transporting layer, and emission layer were fabricated using iridium complex as emission material by solution process. The addition of injection layer was used to increase the device stability and hole injection. The emission layer consisted of phosphor dye and mixed polymer host which blended hole and electron transporting materials. High efficiency and wide emission spectra organic polymer devices with different doping concentrations were fabricated and characterized.

## 2. Experiments

The PLEDs fabrication was described as follows: substrates coated with indium tin oxide (ITO) were first cleaned with water and organic solvents and then underwent surface treatment involving oxygen plasma. This was followed by coating a 50 nm thick layer of poly(ethylene dioxythiophene):poly(styrene sulfonic acid) (PEDOT:PSS) spun on ITO films at 4000 rpm. And then 40 nm thick conductive polymer PVK acting as hole transporting layer was spun on the substrate surface. Next, blended host polymer with Ir(III) complex was spin-coated at room temperature under ambient conditions from a CHCl_3_ solution, resulting in 80 nm thick films. The blended conductive polymer host, poly(9,9-di-n-octylfluorene-2,7-diyl) (PFO) + 30 wt% 2-(4-*tert*-butylphenyl)-5-(4-biphenylyl)-1,3,4-oxadiazole (PBD), was obtained from a commercial supplier and was used as host material without purification. The phosphorescent dye bis[4-tert-butyl-1-phenyl-1H-benzimidazolato-*N*,C^2′^] iridium(III) (acetylacetonate) [(tpbi)_2_Ir(acac)] was synthesized in our lab and used to fabricate small molecular organic device with high efficiency [18]. Finally, a 4 nm thick Ba metal and 150-nm-thick aluminum cathode layer were deposited on the substrate in high vacuum environment through a shadow mask with 5 mm width openings. The devices thus obtained have a typical structure of ITO/PEDOT:PSS (50 nm)/PVK (40 nm)/PFO + 30 wt% PBD:(tpbi)_2_Ir(acac) (*x*%) (80 nm)/Ba (4 nm)/Al (150 nm) (*x* = 1, 2, 4, and 8 for device A, B, C, and D, resp.).

The metal layer thicknesses were determined *in situ* by oscillating quartz thickness monitors and an ellipsometer for the evaporated and spin-coated films, respectively. EL spectra and Commission International De L'Eclairage (CIE) (1931) coordinates of the devices were measured with a PR650 spectroscan spectrometer. Luminance (*L*), current density (*J*), and bias voltage (*V*) characteristics were recorded simultaneously with the measurement of EL spectra by combining the spectrometer with a Keithley 2400 programmable voltage-current source.

## 3. Results and Discussions

**Figure 1:**
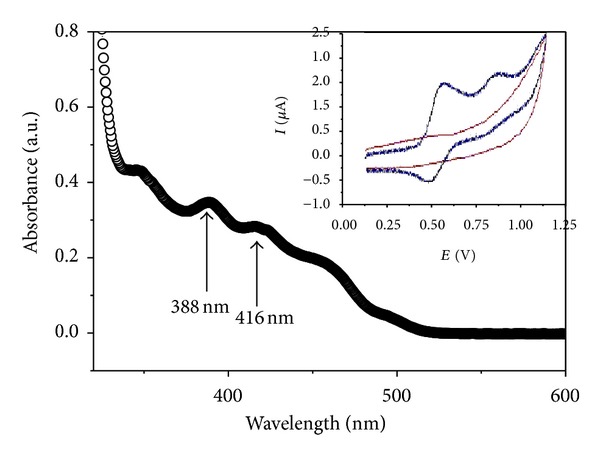
Absorption spectra of (tpbi)_2_Ir(acac) material. The inset was cyclic voltammetry curve of the iridium dye.

**Figure 2:**
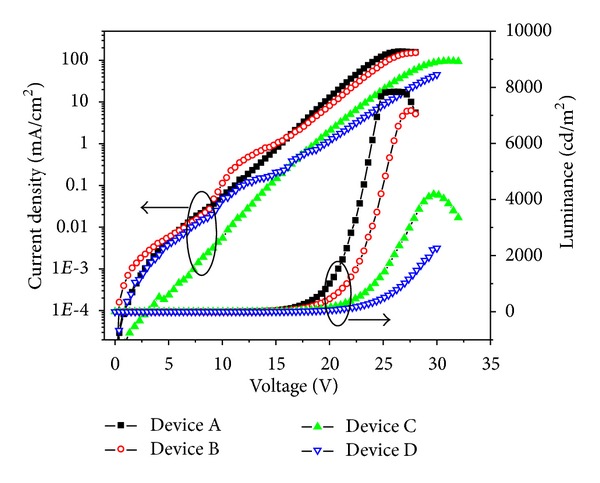
Current density-voltage-luminance characteristics of four doping concentration devices.

In order to indentify the energy transferring from blended polymer host to the doped (tpbi)_2_Ir(acac), the absorption spectra and cyclic voltammetry curve of iridium dye were displayed in Figure 1. There were two absorptive peaks at 388 nm and 416 nm, which were covered by the photoluminescence spectra of mixed PBD:PFO, which indicated the feasibility of energy transfer from host to doped dye. From the absorption and cyclic voltammetry curve, the highest occupied molecular orbital (HOMO) and lowest unoccupied molecular orbital (LUMO) energy level were 5.1 eV and 2.7 eV, respectively. The current density-voltage-luminance characteristics of four doping concentration devices were shown in Figure 2. The turn-on voltage (defined as the voltage when organic device luminance reached 1 cd/m^2^) of 1%, 2%, 4%, and 8% doping concentration devices were 10.0 V, 9.8 V, 12.4 V, and 12.2 V, respectively. The lower doped devices (1% and 2%) showed higher current density than the heavy doped devices (4% and 8%) at the same driving bias. The shift of current density-voltage curves to higher voltages as doping concentration increasing could be attributed to charge-trapping effect [19]. When more iridium complex was doped in host material, more trapping center formed for the free carriers because the HOMO and LUMO level of (tpbi)_2_Ir(acac) were between that of PFO and PBD blended host, so that the current density of device became lower at the same driving voltage when increasing the doping concentration. As the forward bias increased (over turn-on voltage), the luminance of organic devices enhanced sharply and reached to the maximum values of 7841 cd/cm^2^ (at 25.6 V), 7171 cd/cm^2^ (at 27.2 V), 4180 cd/cm^2^ (at 29.6 V), and 2264 cd/cm^2^ (at 30 V) for devices A, B, C, and D, respectively. The device luminance changing trends of different doping concentration devices were in conformity with the current density, which was that higher doping concentration organic device showed lower device luminance at the same bias. The more the doped phosphor dye in the polymer layer, the more the traps or lacunas formed in the organic device, which would capture more charge carriers and affect the emission process of device.

**Figure 3:**
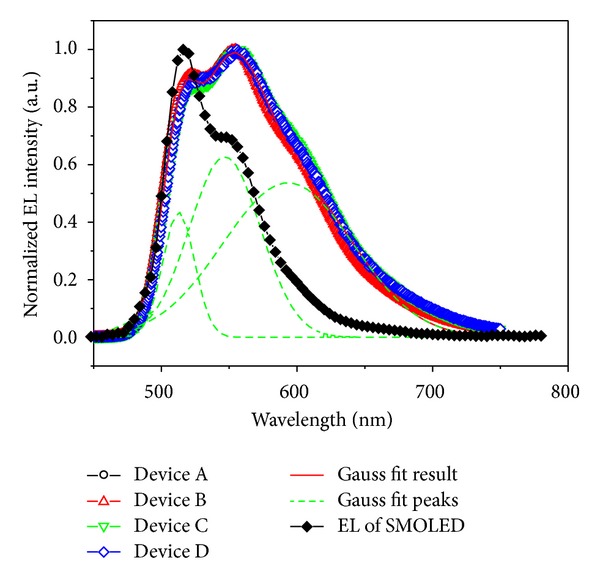
The normalized EL spectra of devices A, B, C, and D at 20 V and Gauss fit results. EL spectrum of SMOLED was shown for comparing.

**Figure 4:**
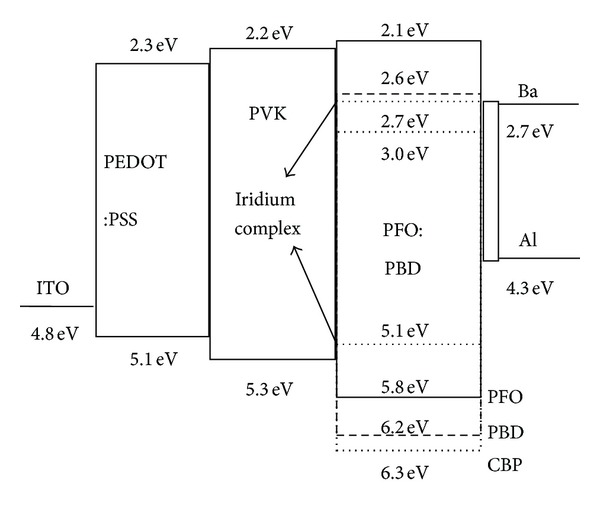
Energy level diagram of polymer light emitting device fabricated in this study.

The normalized EL spectra of polymer devices A, B, C, and D at 20 V bias were suggested in Figure 3. There were two obvious emission peaks around 522 nm and 554 nm, which were attributed to the emission of (tpbi)_2_Ir(acac). The emission peak coming from phosphor dye could be identified by small molecular organic light-emitting diodes (SMOLED) with CBP as host material. The SMOLED doped by the phosphor dye had been fabricated with device structure ITO/CuPc (40 nm)/*α*-NPD (45 nm)/CBP:(tpbi)_2_Ir(acac) (3 wt%, 30 nm)/BCP (20 nm)/Alq_3_ (20 nm)/LiF (1 nm)/Al (100 nm), and EL spectrum of the device was also shown in Figure 3 for comparing [18]. But the strongest emission peak was changed from 522 nm to 554 nm for polymer device, and the spectrum width of PLED was nearly double that of SMOLED. The spectrum of polymer light-emitting diodes covered from 490 nm to 690 nm, and full width at half-maximum (FWHM) of emission spectrum was 116 nm (from 504 nm to 620 nm), which was 68 nm for SMOLED (from 500 nm to 568 nm). There was an obvious spectra extension at the long wave region (~600 nm), which might be caused by the blended polymer host materials. To describe the phenomenon clearly, energy level of polymer device was shown in Figure 4. In small molecular organic device with (tpbi)_2_Ir(acac) doped in CBP host, the HOMO and LUMO energy level of iridium complex and CBP were 5.1 eV and 2.7 eV, 6.3 eV, and 3.0 eV, respectively. (tpbi)_2_Ir(acac) molecules could be excited by those excitons with energy transfer from CBP host, but the kinds of excitons formed in CBP were simplex because of single energy level structure. In the blended polymer host structure of this research, the HOMO and LUMO energy level of PFO and PBD were 5.8 eV and 2.1 eV, 6.2 eV, and 2.6 eV, respectively. The HOMO and LUMO energy level of (tpbi)_2_Ir(acac) could be identified by the cyclic voltammetry curve as shown in the inset of Figure 1. After doping iridium complex in the blended polymer host, the phosphor dye could be excited by trapping the free carriers directly in the host layer and accepting energy transfer from host excitons. The blended polymer host promoted the energy transferring from different energy level to phosphorescent dye, which could extend the emission spectrum to yellow light region width of polymer emission device. The Gaussian multipeak simulation indicated that there were three peaks (513 nm, 548 nm, and 596 nm) in the phosphor emission spectrum, and the total fitting spectrum curve was highly consistent with the test result as shown in Figure 3. The emission peak at 596 nm occupied near 50% of the total emission spectra area, which indicated this energy level accepted about 50% energy transfer from polymer host and formed effective light emission.

PEDOT:PSS layer spun on ITO films was used to improve the substrate smoothness, and the other important role was that this film has approximate work function with ITO anode [20], which would be good for hole injection due to the lower height of injection barrier. PVK was a very good hole transporting and wide band gap material but not an electron transporting material [21], so that PVK could be used as hole transporting layer with blocking electronic function. PVK was also a high molecular weight material, which was helpful in forming thin dense films with high uniformity and improving the film-forming quality for the emitting layer and finally improving the stability of device. The HOMO and LUMO level of PFO were estimated to be 5.8 eV and 2.1 eV [22], so that the wide band gap (3.7 eV) of PFO made it difficult to form ohm contacts for free carriers injecting into emission layer, which was unsuitable to obtain high efficiency device. In order to decrease the LUMO energy level barrier and promote electron transporting ability, 30 wt% PBD was mixed to form blended host matrix. PBD was widely used as electron transporting layer, which showed the HOMO and LUMO level of 6.2 eV and 2.6 eV, respectively [23]. The LUMO level of PBD was very near to the work function of Ba layer, which was inserted between PFO:PBD blended layer and Al cathode with a super-thin film thickness. The 4 nm Ba film promoted the injection of electrons from cathode to host layer to be much easier than that directly from Al metal (work function, 4.7 eV). The addition of PVK transporting layer, PFO:PBD blended host layer, and Ba buffer layer was propitious to increase the stability and efficiency of polymer device.

**Figure 5:**
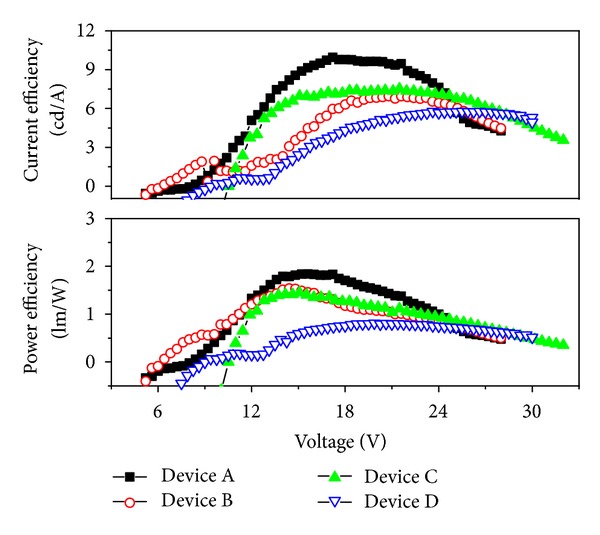
The power efficiency and current efficiency of four doping devices at different biases.

The power efficiency (*η*_*p*_) and current efficiency (*η*_*l*_) of four doping concentration devices (device A: 1 wt%, device B: 2 wt%, device C: 4 wt%, and device D: 8 wt%) at different biases were shown in the Figure 5. Both *η*_*p*_ and *η*_*l*_ initially increased to the maximum value and then decreased as the bias voltage increased for those polymer devices. The maximum *η*_*l*_ of device A, B, C, and D were 9.95 cd/A (at 17.2 V), 6.96 cd/A (at 20.8 V), 7.52 cd/A (at 21.4 V), and 5.78 cd/A (at 26.2 V), respectively. The maximum luminance and power efficiency of four devices (shown in Table 1) became lower when increasing the doping concentration. The doping phosphor dye was not only the emission center but also traps for free carriers in emission layer, which could be predicted from the device energy level diagram shown in Figure 4. More free carriers would be trapped in the devices when adding more phosphor dye in the blended host layer, which would affect the current density, numbers of emission elements, and finally the whole device efficiency. This point could be proved by the current density curve of four devices at high driving voltages shown in Figure 2. The current density of device A (1 wt% doping concentration) was 12.8 mA/cm^2^ and decreased to 1.2 mA/cm^2^ for device D (8 wt% doping concentration) at 20 V forward bias, which were listed in Table 1. The roll-off of external quantum efficiency of electroluminescent device at large current density was due to triplet-triplet (T-T) annihilation for most phosphorescent organic device. Endo et al. analyzed T-T annihilation for Ir(ppy)_3_ doped CBP host devices, and a best fit of the model to the data was obtained [24]. Almost all organic devices indicated a gradual decrease in efficiency at the high current density (according to high voltage). The quantum efficiency of light emission (*η*) can be calculated from the following equation:

(1) $$\begin{array}{c} \frac{\eta }{\eta_{0}}=\frac{J_{0}}{4J}\left(\sqrt{\frac{1+8J}{J_{0}}}-1\right). \end{array}$$

In (1), *η*_0_ is the quantum efficiency without triplet-triplet annihilation, and *J*_0_ in (2) is the “onset” current density at *η* = *η*_0_/2:

(2) $$\begin{array}{c} J_{0}=\frac{4qd}{k_{\text{TT}}\tau^{2}}. \end{array}$$

In which *q* is the electron charge, *d* is the thickness of the exciton formation zone, *τ* is the phosphorescent life time, and *k*_TT_ is the T-T annihilation rate constant. As the current density (driving bias) increased, the efficiency annihilation became more obvious as shown in (1). For comparing, the turn-on voltage (*V*_*on*⁡_), maximum lumiance (*L*_max⁡_), current density at 20 V (*J*_20 V_), maximum power efficiency (*η*_*p*max⁡_), and current efficiency (*η*_*l*max⁡_) were list in Table 1. Stable green light emission, wide emission spectra, and high efficiency of 9.95 cd/A could be achieved with the 1 wt% doping concentration polymer device with blended host materials.

## 4. Conclusions

Different doping concentration polymer organic devices with iridium complex doped in PFO:PBD blended host material were fabricated using spun coat process. The EL spectra of devices indicated stable green light emission from (tpbi)_2_Ir(acac) with two main peaks at 522 nm and 554 nm and a wide FWHM of 116 nm from 504 nm to 620 nm. Maximum current efficiency of 9.95 cd/A could be reached at 17.2 V bias and slowly rolled off as the driving voltage increased. High quality, easy fabricated white light emission polymer device could be anticipated with this wide emission spectra device structure.

## Figures and Tables

**Table 1:** Some detailed characteristics of four organic devices with different doping concentrations, including turn-on voltage, maximum luminance, current density at 20 V, maximum current, and power efficiency.

Device	*V*_*on*⁡_ (V)	*L*_max⁡_ (cd/m^2^)	*J*_20 V_ (mA/cm^2^)	*η*_*p*_max⁡__ (lm/W)	*η*_*l*_max⁡__ (cd/A)
Device A	10.0	7841	12.8	1.84	9.95
Device B	9.8	7171	8.2	1.54	6.96
Device C	12.4	4180	2.1	1.44	7.52
Device D	12.2	2264	1.2	0.80	5.78
